# CAMERA: A Community Resource for Metagenomics

**DOI:** 10.1371/journal.pbio.0050075

**Published:** 2007-03-13

**Authors:** Rekha Seshadri, Saul A Kravitz, Larry Smarr, Paul Gilna, Marvin Frazier

## Abstract

The CAMERA (Cyberinfrastructure for Advanced Marine Microbial Ecology Research and Analysis) community database for metagenomic data deposition is an important first step in developing methods for monitoring microbial communities.

**Figure:**
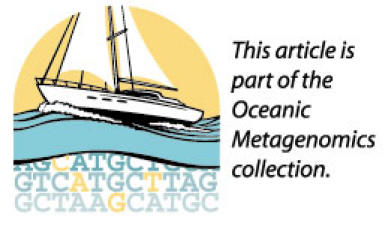


Microbes are responsible for most of the chemical transformations that are crucial to sustaining life on Earth. Their ability to inhabit almost any environmental niche suggests that they possess an incredible diversity of physiological capabilities. However, we have little to no information on a majority of the millions of microbial species that are predicted to exist, mainly because of our inability to culture them in the laboratory.

A growing discipline called metagenomics allows us to study these uncultured organisms by deciphering their genetic information from DNA that is extracted directly from their environment, thus effectively bypassing the laboratory culture step. Metagenomics allows us to address the questions “who's there?”, “what are they doing?”, and “how are they doing it?”, offering insights into the evolutionary history as well as previously unrecognized physiological abilities of uncultured communities.

Studies such as the J. Craig Venter Institute's Global Ocean Sampling (GOS) expedition (in this issue) reveal a remarkable breadth and depth of microbial diversity in the oceans. To date, researchers have made significant but largely preliminary inroads into understanding the biogeography of microbial populations across ecosystems. We know even less about the dynamic physiological processes and complex interactions that impact global carbon cycles and ocean productivity. Marine microbes are thought to act as part of the biological conduit that transports carbon dioxide from the surface to the deep oceanic realms. By removing carbon from the atmosphere and sequestering it (in the form of organic matter), marine microorganisms may significantly affect global climate. Although we now have numerous global and real-time methods to measure physical and chemical parameters within the ocean, few methods or concepts have been developed to measure important microbial processes on a global scale. Even if the technology to make such measurements existed, we would presently not know what to measure or how to interpret those measurements.

We invite the research community to submit its metagenomics data to CAMERA.

We need a systematic way to explore the structure and function of ocean ecosystems, and their impact on global carbon processing and climate. Metagenomics has the potential to shed light on the genetic controls of these processes by investigating the key players, their roles, and community compositions that may change as a function of time, climate, nutrients, carbon dioxide, and anthropogenic factors. These studies include a substantial informatics component, requiring researchers to take on complex computational and mathematical challenges. Nonetheless, microbiologists have been quick to seize upon this modern technique, resulting in a deluge of sequence data, and an ever-widening gap between the rates of collecting data and interpreting it.

The Community Cyberinfrastructure for Advanced Marine Microbial Ecology Research and Analysis (CAMERA) project [1] is an important first step in attempting to bridge these gaps and in developing global methods for monitoring microbial communities in the ocean and their response to environmental changes. The aim is to create a rich, distinctive data repository and bioinformatics tools resource that will address many of the unique challenges of metagenomics and enable researchers to unravel the biology of environmental microorganisms (Figure 1). CAMERA's database includes environmental metagenomic and genomic sequence data, associated environmental parameters (“metadata”), precomputed search results, and software tools to support powerful cross-analysis of environmental samples.

**Figure 1:**
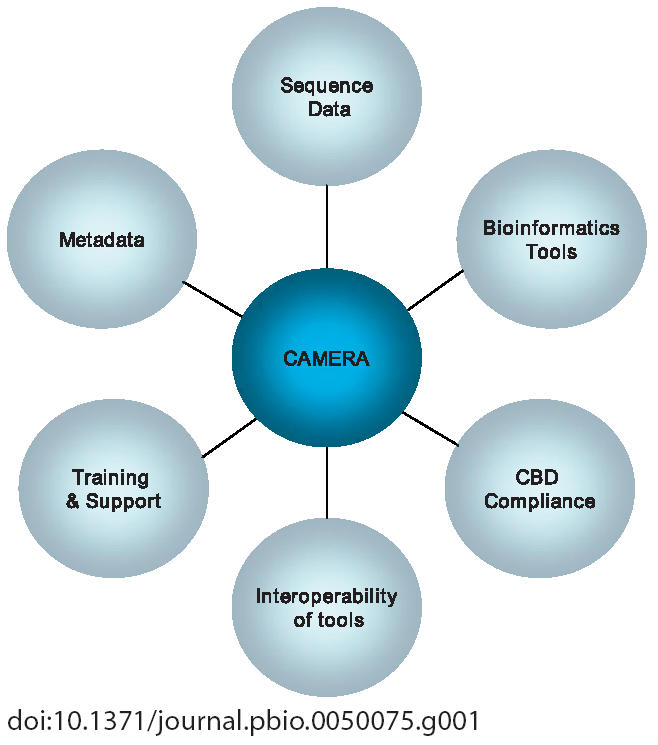
Schematic of Intended Core Functions of the CAMERA Project CBD, Convention on Biological Diversity.

The initial release will include data and tools associated with the companion set of GOS expedition publications [2–4]; metagenome data from the Hawaii Ocean Time Series Station ALOHA [5] and marine viromes from four different oceanic regions[6]; standard nonredundant sequence databases (e.g., nrnt for nucleotides and nraa for amino acids[7]); and collections of microbial genome sequences, including a set of 155 marine microbial genomes funded by the Gordon and Betty Moore Foundation. The focal point for the CAMERA project is its Web site: http://camera.calit2.net. We invite the research community to submit its metagenomics data to CAMERA, and are establishing mechanisms to streamline this process. Here we describe some of the key challenges and features of the CAMERA project.

## Accessibility of Metadata

Existing data repositories provide limited support for metadata and metadata-based queries—including any supplemental information for the sequence data, such as pH and temperature of water at the collection site—and therefore these metadata go underutilized by the research community. CAMERA will integrate sequence data with all available, relevant metadata, including physical information (e.g., temperature and sample method), chemical information (e.g., salinity and pH), temporal information, geospatial information, methodology and instrumentation used for data collection, and satellite images of the collection site. These contextual data allow researchers to derive correlations between deciphered ecology and the environmental conditions that may favor one community structure over another. One can envision a future where metadata from satellites and weather stations, and other physicochemical data, can be used to help interpret and inform scientists on how these factors affect microbial processes as well as community composition. CAMERA is working with other groups (e.g., Genome Standards Consortium) to establish standards for the information content and format of metagenomic data and metadata submissions.

## New-Generation Bioinformatics Tools

**Figure 2:**
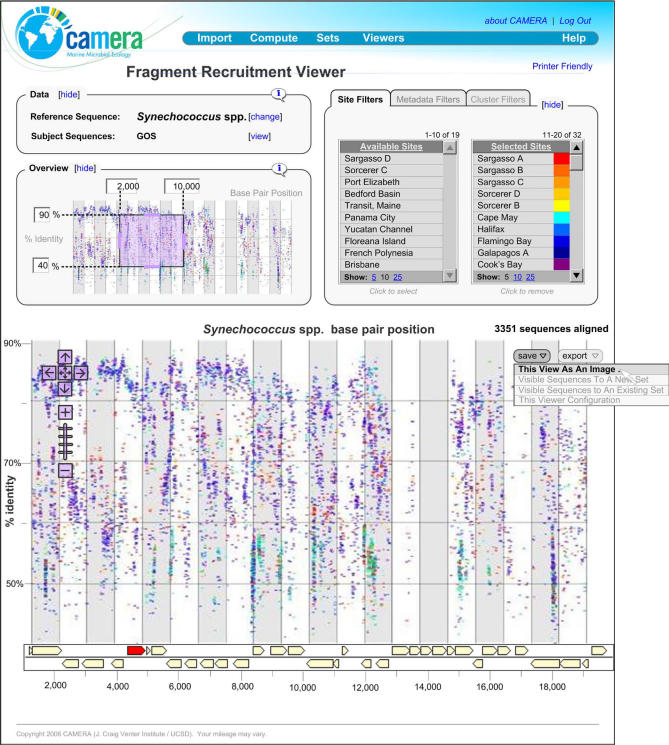
CAMERA Fragment Recruitment Viewer This tool graphically displays the results of a BLASTN sequence comparison of an available microbial genome against selected sequence read datasets. The example shown displays the abundance and distribution of Synechococcus spp. genome sequence in the selected sampling sites. The Synechococcus spp. genome coordinates are shown on the *x*-axis, while the *y*-axis shows the percent identity scores of the alignment to the selected Sargasso Sea and GOS sequence reads. The viewer incorporates metadata associated with the reads, allowing a user to quickly identify data of interest for further examination. The utility of the plot is to examine the biogeography and genomic variation of abundant microbes when a close reference genome exists.

Analysis and comparison of complex metagenomic data is driving the development of a new class of bioinformatics and visualization software. CAMERA will integrate these tools with its database, couple them with large-scale compute resources, and make them widely available to the research community. Initially, CAMERA will support analytical tools used for analyses in the GOS publications [2–6]. An example is shown in Figure 2: a subset of metagenome sequence reads from GOS environmental samples is compared to a reference genome sequence (Synechococcus spp.) using BLASTN. The results and underlying metadata are displayed through an interactive graphical viewer, which helps users quickly identify sequence reads that are similar to the reference genome sequence, and potentially identify metabolic similarities between microbes in environmental samples and a reference microbe. A detailed description of this tool and its applications are provided in the GOS companion paper by Rusch et al. [2]. CAMERA will work closely with the community to identify and incorporate additional tools and workflows.

## Large-Scale, Robust, and Expandable Cyberinfrastructure

The enormousness of metagenomics datasets requires terascale computation and storage facilities. CAMERA is building a state-of-the-art computational infrastructure to provide high-performance networking access and grid-based computing (applying the resources of many computers in a network to a single problem at the same time), and to support new ways of visualizing and interacting with the data. The distributed architecture of the CAMERA computational engine will be based on the National Science Foundation–funded OptIPuter project [8,9], which allows for use of dedicated 1- or 10-Gbps optical fiber links between remote user laboratory clusters and the CAMERA compute complex. The data server complex itself will contain a large amount of rotating storage (ultimately several tens of terabytes replicated) and a large computational cluster (upwards of a thousand processors). It will be augmented on demand by a scalable back end provided by the recently upgraded National Science Foundation TeraGrid.

## Recognition of the Sources of Samples

The Convention on Biological Diversity grants countries certain rights over their genetic resources, including, for example, metagenomic sequence data of marine microbes taken from a country's territorial waters. Many countries require, at minimum, that databases explicitly identify the country of origin of the DNA. Rules vary by country, and it is not a simple task to find out what might be required. International harmonization of these rules is currently being debated by the over 150 countries that are party to the Convention on Biological Diversity. Agreements about the use of genetic resources are negotiated on a case-by-case basis with each researcher who wishes to sample within a country's “exclusive economic zone,” typically 200 miles from its shoreline. Some of these “memoranda of understanding” impose additional requirements on the researchers. For example, the J. Craig Venter Institute's agreement with Australia requires us to “use reasonable effort to notify Australia as soon as possible of any inquiries for commercial purposes.”

Current databases do not allow the original investigators to inform others about the details of an agreement, thus creating a significant roadblock to both the collection and public release of metagenomics data. To address this issue, CAMERA data will only be made available to users who register by supplying a suitable E-mail address and who acknowledge the potential restriction on commercial use by countries from which the data were collected. To further comply with the Convention on Biological Diversity, all data objects served by CAMERA will possess a mapping to the country of origin of the underlying DNA sample.

## Outreach and Training

Since the ultimate success of CAMERA will depend on the broader research community's ability to make use of the novel cyberinfrastructure, a series of on-site and Web-based training programs will be provided to keep users apprised of CAMERA's functionalities and to support integration of CAMERA's service-oriented architecture into their computational fabrics. Finally, we envision interacting with the community on several fronts, including standardization of ontology, metadata, nomenclature, and tools, and incorporation or federation of existing tools and resources with CAMERA.

We believe that the data and community cyberinfrastructure provided by CAMERA will help researchers to advance understanding of the codependence or feedback between microbial communities and biogeochemical processes in oceans over time, and of how perturbations in the environment cause compositional changes (including extinction). Eventually, the expanded global environmental metagenomics datasets will enable better monitoring of environmental change and the processes that control climate. Systematic and routine monitoring of genomic signatures of global microbial populations and processes overlaid with meteorological information and other metadata may help researchers explain past shifts in global climate as well as predict future changes. This knowledge may someday guide decisions about acceptable atmospheric levels of greenhouse gases, or guide strategies to increase sequestration of atmospheric carbon dioxide by changing ocean microbial compositions, in order to reverse the effects of global warming.

## Abbreviations

CAMERA: Community Cyberinfrastructure for Advanced Marine Microbial Ecology Research and Analysis
GOS: Global Ocean Sampling
